# Investigation of Topographical Alterations in Titanium-Zirconium-Alloy Implant Threads following Er:YAG Irradiation

**DOI:** 10.3390/ma15227889

**Published:** 2022-11-08

**Authors:** Mustafa Ayna, Johannes Spille, Yahya Açil, Jan-Tobias Weitkamp, Jörg Wiltfang, Cemal Esen, Aydin Gülses

**Affiliations:** 1 Department of Periodontology, Bonn University, 53111 Bonn, Germany; 2 Department of Oral and Maxillofacial Surgery, Christian Albrecht University, 24105 Kiel, Germany; 3 Laser Technology, Ruhr University, 44801 Bochum, Germany

**Keywords:** Er:YAG, dental, peri-implantitis, roughness, titanium-zirconium alloy

## Abstract

The aim of the current experimental study was to comparatively assess the surface alterations in titanium and titanium-zirconium alloy implants in terms of thread pitch topography after irradiation with an Er:YAG laser, which is recommended in the literature for its sterilizing effect in the treatment of contaminated implant surfaces. Roxolid^®^ and SLA^®^ (Sand-blasted, Large-grit, Acid-etched) implants from Straumann^®^ company with the same macro topography were investigated. The surface treatment was carried out using a wavelength of 2940 nm, 60 s irradiation time, a frequency of 10 Hz, and energies between 120 mJ and 250 mJ. The alterations were quantitatively analyzed by conducting roughness analysis via white light interferometry and qualitatively using SEM images. Roxolid^®^ could particularly maintain its surface topography at a level of 160 mJ. At an energy level of 250 mJ, the surface properties of the pitch could be significantly altered for the first time. Compared to the Standard Plus dental implants studied, no distinct removal of the material from the surface was detected. The alloy properties of Roxolid^®^ confirm the manufacturer’s statement in terms of stability and could offer advantages in peri-implantitis management if decontamination has been selected. However, as a part of a respective strategy, smoothening of a Roxolid^®^ implant surface requires a significantly higher energy level compared to SLA-Standard^®^ dental implants.

## 1. Introduction

Within the last two decades, implant therapy has become a routine treatment option in daily dental practice. However, recent epidemiological studies have shown average prevalence rates of 43% and 22% for inflammation-related diseases such as mucositis and peri-implantitis, respectively [1]. In the literature, it has been well described that peri-implant mucositis can be treated effectively with mechanical non-surgical therapy using debridement and/or antimicrobial therapy; however, peri-implantitis management often requires a surgical intervention to reduce the bacterial load and eliminate the peri-implant pockets, decontaminate implant surfaces, and, in some cases, attempt to bring about bone regeneration afterward [2]. It is also critical that the exposed implant surfaces can be easily managed within the daily oral hygiene routine of the patients. Consequently, various surgical approaches have been proposed, including resective and regenerative procedures, such as implantoplasty and implant surface decontamination, to ensure smooth surfaces. Moreover, adjunctive therapy options using photodynamic therapy, chemical decontamination, or cold atmospheric plasma have been also proposed [3,4].

Laser treatment can be applied as a monotherapy and as an adjunct to the surgical and non-surgical treatment of peri-implantitis. The clinical efficiency of different laser applications in peri-implantitis therapy has been widely discussed [5]. CO_2_ lasers are considered to be safe and able to enhance bone regeneration. The diode laser seems to be effective in its bactericidal effect without changing the implant surface pattern. The erbium/chromium-doped yttrium-scandium-gallium-garnet (Er,Cr:YSGG) laser can also stimulate bone regeneration, and the Erbium-doped yttrium aluminum garnet (Er:YAG) laser exhibits a strong bactericidal effect against periodontopathic bacteria at a low energy level. The Er:YAG laser is a good opinion for dental applications because of its 2940 nm wavelength, which is highly absorbed by water. This enables a sterilizing effect in the treatment of contaminated implant surfaces. In the literature, positive effects are described in terms of the bactericidal property, mainly obtained using the Er:YAG laser [6].

Most of the studies on peri-implantitis have focused on the management of titanium/titanium alloy implants; thus, the main alloys are so-called “commercially pure” titanium and Ti-6Al-4V, both of which yield clinical success rates of up to 99% at 10 years [7] Besides those, different Ti-alloys such as TiAl_6_Nb_7_ could also be indicated to allow provisional solutions and used for the production of one-piece transitional implants, which are elements that are basically designed and manufactured to ensure the fixation of fixed and removable temporary dentures. This therapy method is especially of interest to patients with high expectations since, despite the establishment of the new concepts in immediate or early loading protocols, an immediate prosthetic restoration cannot always be carried out. As a result of the developments in materials science, definitive outcomes can also be expected from temporary implants. However, the dental clinician should keep in mind that the over all treatment goal of transitional implants is not osseointegration but rather to serve as auxiliary elements for temporary restorations [8]. Nowadays, another clinical application for Ti-alloys especially for grade V titanium is their use for the production of custom-made implants using laser sintering (SLM/Selective Laser Melting) technology according to the DICOM (Digital Imaging and Communications in Medicine) files from computed tomography using computer-aided design and segmentation software [9]. This technique could avoid excessive bone augmentation in well-selected cases and also allow immediate functional loading. Despite the ongoing dominance of Ti alloys in dental implantology, various materials such as zirconia, zirconium alloy, or peek were also used in dental implantology. A relatively novel alloy that might present higher resistance to mechanical stress compared to the pure titanium used nowadays to produce standard dental implants is the Roxolid^®^ implant (Institut Straumann AG, Basel, Switzerland) of titanium–zirconium alloy (83–87% titanium, 13–17% zirconium). Several biomechanical tests on experimental models have shown a higher resistance to loading stresses compared to the standard, pure titanium implants. However, the number of studies focusing on the effects of a laser on Roxolid^®^ surfaces is limited.

In addition to their superior mechanical properties regarding tensile strength [10], the surface characteristics such as roughness, surface charge, or hydrophilicity of Ti alloys play a key role in their biocompatibility, which has a direct impact on clinical outcomes [11]. Additionally, Teughels et al. have shown that the roughness of the implant surface also has a significant impact on the quantity and quality of the plaque formed. Thus, rough surfaces and those presenting a greater surface free energy lead to more plaque accumulation [12]. Furthermore, initial bacterial adhesion starts in areas of high wettability and inside the pits and grooves of the roughened surfaces, from where it is difficult to eliminate it [13]. Romeo et al. have also shown that obtaining smooth surfaces after peri-implantitis therapy could improve the prognosis for the affected implant [14].

Roughness parameters used for the evaluation of implant surfaces can vary. White light interferometry is a well-known measurement technique commonly used in the 3D shape and roughness characterization of engineered and biological objects and is often recommended for measuring the roughness of titanium and or titanium/alloy surfaces [15,16].

The evidence regarding the long-term benefits of lasers in the treatment of peri-implant inflammation is controversial [4]. In the literature, numerous articles have investigated the effects of laser irradiation in both the resective and regenerative management of Ti-surfaces, which have mostly focused on Er:YAG, CO_2_, or Nd:YAG lasers [17]. However, not a single article has examined the ablative effects of Er:YAG irradiation on titanium-zirconium alloys.

The Er:YAG laser has been demonstrated to have an excellent ability to ablate both hard and soft tissues [18]; however, there are controversies regarding the effectiveness of the use of the Er:YAG laser in the treatment of peri-implantitis [19]. A recent study has shown that Er:YAG lasers could effectively reduce the probing depth and gingival recession in patients with peri-implantitis [20]. Therefore, there is a need for research emphasizing a detailed description of the specific laser’s characteristics and power settings in various implant surfaces [21]. The aim of the current experimental study was to comparatively assess the surface alterations of titanium and titanium-zirconium alloy implants in terms of thread pitch topography after irradiation with an Er:YAG laser. To the best of our knowledge, the current paper is the first study in the literature in English that has evaluated the effects of the Er:YAG laser on Roxolid^®^ surfaces.

Existing investigations of the Er: YAG laser have shown its promising potential in the management of peri-implantitis therapy [20] and gave the impetus for the present paper, where the Er:YAG laser is applied on SLA and Roxolid^®^ surfaces. Current emphasis is placed on the ablative effects of an Er:YAG laser in different wavelengths, which might be used in the resective treatment of peri-implantitis.

## 2. Materials and Methods

### 2.1. Study Design and Selection of the Implants

**Figure 1:**
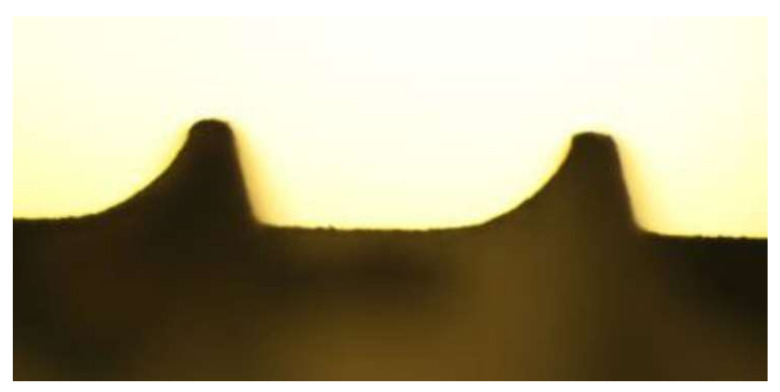
2.5× magnification with the microscope TMS-1200 TopMap µ.Lab (Institut Polytec, Waldbronn, Germany) of the threads of the SLA-Standard implant from the Straumann Company.

**Figure 2:**
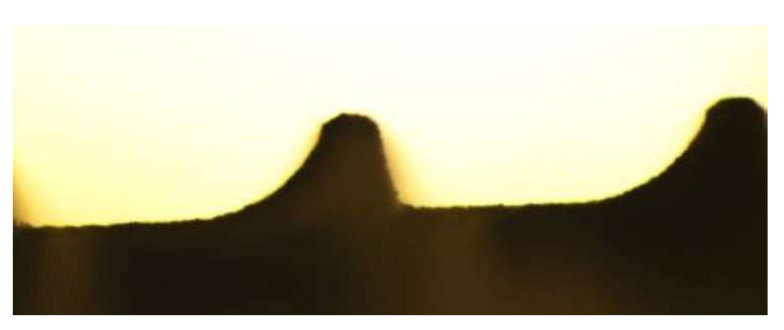
2.5× magnification with the microscope of the threads of the Roxolid implant from the Straumann Company.

Two different implant systems from Straumann^®^ company (Institut Straumann, Basel, Switzerland) with the same macro-topography were investigated:

- SLA^®^ (Sand-blasted, Large-grit, Acid-etched), length 14 mm, diameter 4.8 mm: The surface was sandblasted (grain size 250–500 μm), which produced a macro-roughness of about 20–40 μm between the peaks (peak-to-peak). Due to acid etching, a micro-roughness of about 2–4 μm was achieved. The implant was made purely of titanium. Figure 1 shows an SLA-Standard implant.

- Roxolid^®^, length 14 mm, diameter 4.8 mm: Roxolid is a titanium-zirconium alloy (85% titanium, 15% zirconium). The zirconium percentage makes the implant appear lighter. Figure 2 shows a Roxolid implant.

### 2.2. Laser Application

**Figure 3:**
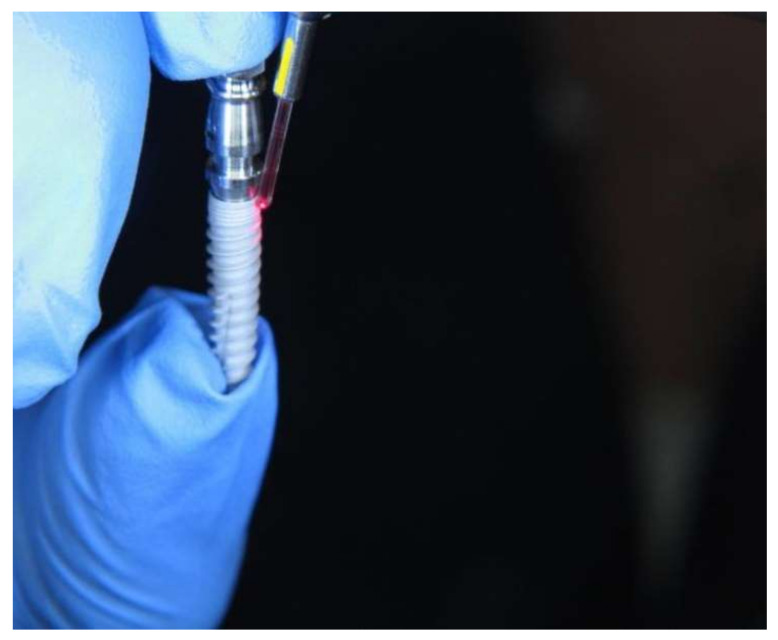
Laser treatment of the implant surface.

The threads of the implants were used starting at the coronary area. The surface treatment was carried out using an Er:YAG Laser (KaVo Key Laser^®^ System Aesculap (Institute KaVo, Biberach, Germany)) (solid state erbium: yttrium, aluminum, garnet) operating at a wavelength of 2940 nm and thermomechanical ablation. (Figure 3) Energies were adjusted at 120 mJ (thread area “1”), 140 mJ (thread area “2”), 160 mJ (thread area “3”), 180 mJ (thread area “4”), 200 mJ (thread area “5”), and 250 mJ (thread area “6”). The irradiation time for all energies was 60 s, and the frequency was 10 Hz. Areas at the respective implant threads without surface treatment were examined as a control group.

**Figure 4:**
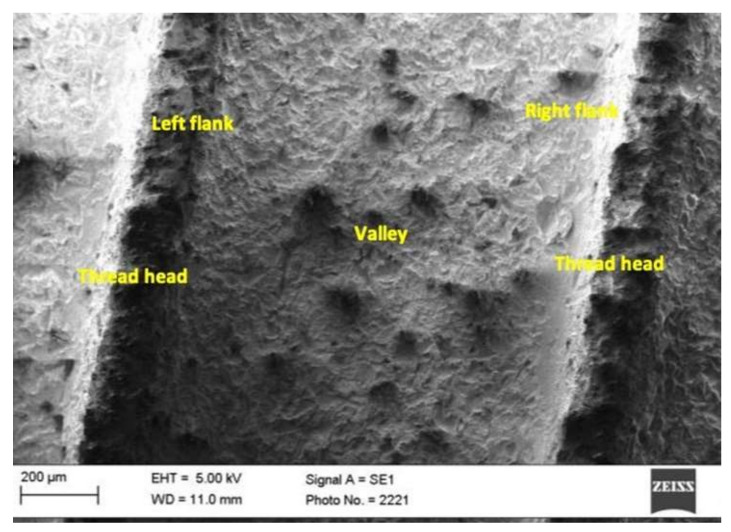
SEM image of an implant, which is divided into different partial surfaces in the thread, such as head, valley, and right and left flanks.

In each implant thread, the partial surfaces of the head, the valley, and the right and the left flanks were irradiated and measured separately (Figure 4). The right flank was described as the coronal part at the thread pitch, whereas the left flank belonged to the apical thread of the pitch evaluated. On each implant, a total of four pitches were treated by the Er:YAG laser. To avoid operator errors, each measurement was repeated three times.

### 2.3. Roughness Analysis

**Figure 5:**
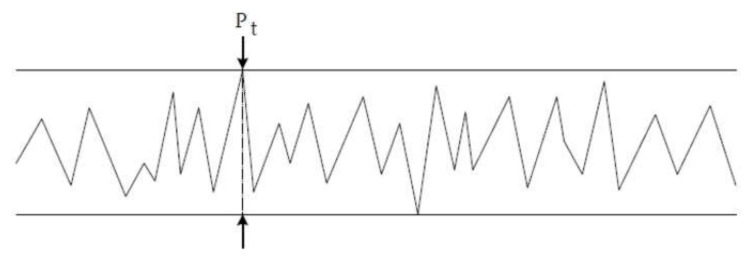
Profile section of profile depth Pt.

**Figure 6:**
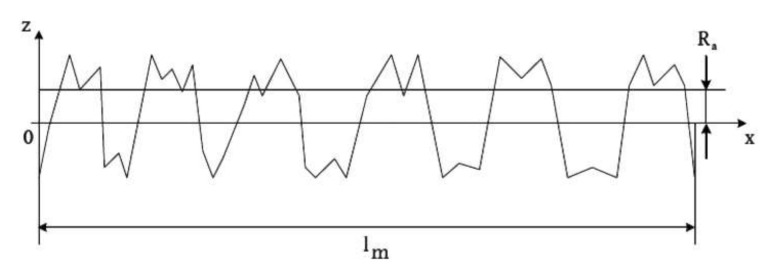
Presentation and calculation of the arithmetic average roughness Ra. Ra shows the roughnesses in the *z*-axis, peaks and grooves are measured, time is represented on the *x*-axis.


Roughness analysis was conducted according to the date executed from the measurement of:

- The Profile depth (Pt) (Figure 5)
- Arithmetic average roughness (Ra) (Figure 6), which was calculated according to
- Mean surface roughness Rz, determined from the arithmetic mean of the individual roughness depths Z1–Z5 of five adjacent individual measuring sections in the roughness profile.

(1) $$R_{Z}=\frac{1}{5}\times \left(R_{Z1}+R_{Z2}+R_{Z3}+R_{Z4}+R_{Z5}\right)$$

**Figure 7:**
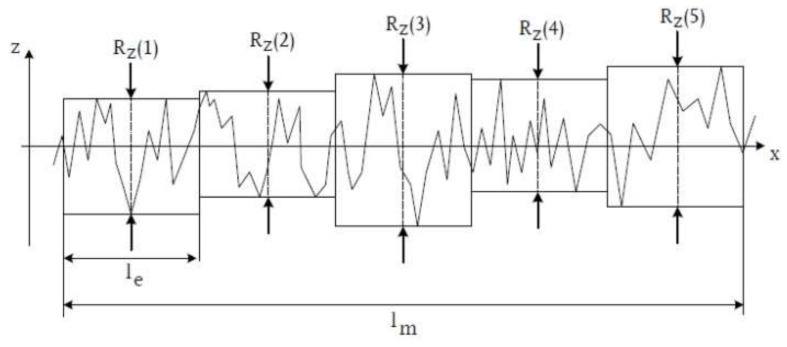
The distance between two parallels to the middle line is the individual peak-to-valley height that meets the maximum or minimum of the roughness profile within the individual measurement section. Thus, the surface roughness Rz is a measure of the average vertical surface fissures. (Rz is determined from the arithmetic mean of the individual roughness depths Z1 - Z5 of five consecutive individual measuring sections. The z axis shows the roughnesses, the x axis shows the time).

These equal distances correspond to the length of the cut-off wavelength (Figure 7).

The white light interferometer TMS-1200 TopMap µ.Lab from Polytec (Polytec Group, Hörsching, Austria) was used to measure the machined and unmachined surfaces of implants. It enables the measurement of structures ranging in size from centimeters to micrometers. For visualization, the white light interferometer was combined with a microscope with 20× magnification.

**Figure 8:**
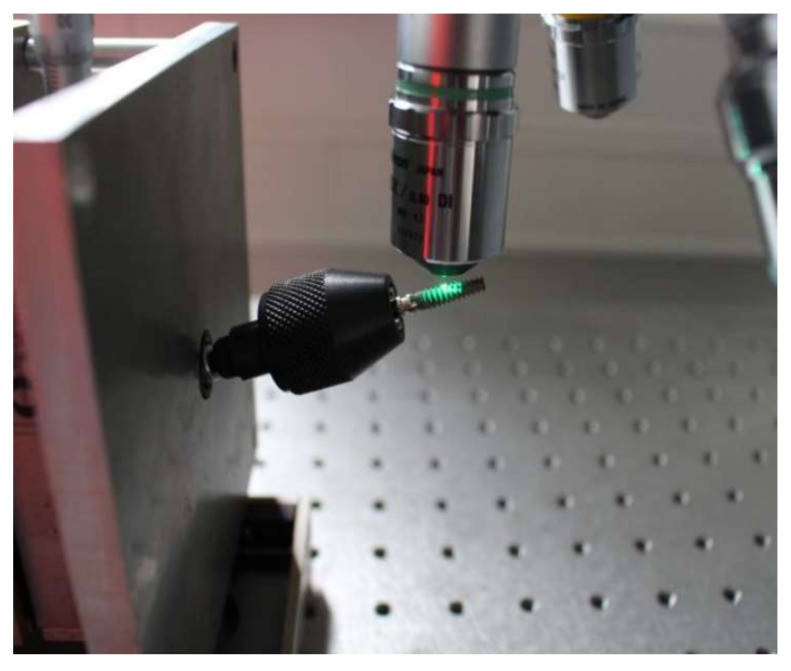
Measurement of implant roughness using white light interferometer connected to the microscope.

In white light interferometry, the height profile—hence the roughness of a measurement object (surface of the implant)—could be determined by exploiting interference effects. The measurement object was placed in one arm of the white light interferometer. The incident light split into two rays at a semi-transparent mirror. One ray fell on the measurement object; the other was reflected toward a reference mirror. After reflection or transmission at the mirror, both rays met again and interfered. The returning rays were transmitted to the CCD sensor (“charge-coupled device”) and formed an interference signal for each individual pixel depending on the position of the measurement object. The implant always had to be placed at a 90-degree angle to the measuring direction (Figure 8).

**Figure 9:**
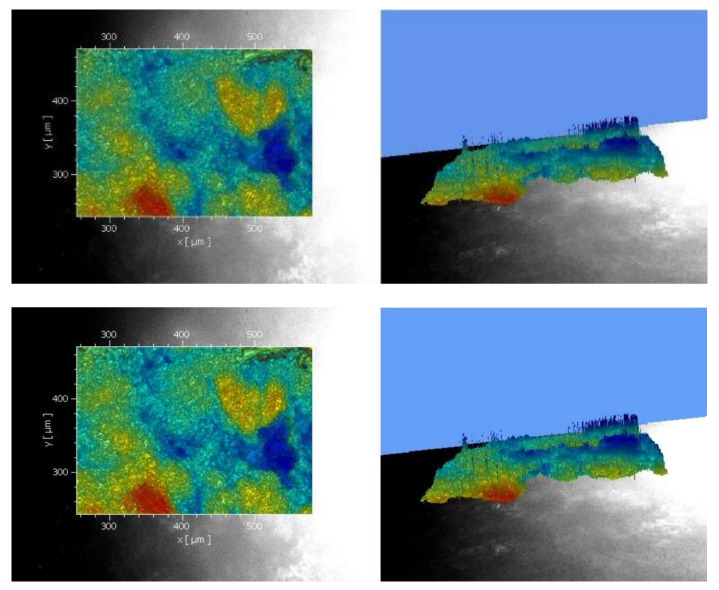
Records of the surface roughness with linear regression, left 2D and right 3D view.

Therefore, the implant position was manually adjusted to measure the different areas. For each sample, a profile section was created, and the values of the profile depth, the mean peak-to-valley height, and the arithmetic mean peak-to-valley height were used for the evaluation (Figure 9). Data regarding the profile section and Ra, Rz, and PT values were executed.

### 2.4. Statistical Analysis

Statistical analyses were performed using the Statistical software SPSS (IBM^®^, Ehningen, Germany). Measurements of implant surfaces using white light interferometer were expressed as arithmetic means. The ratio of untreated and treated implant surfaces (implant areas “1” to “6”) with the Er:YAG laser of different energies was calculated. The relation between variables was evaluated using the Sample *t*-test and Mann-Whitney U test. Associations were considered significant when the *p*-value was < 0.05.

## 3. Results

**Figure 10:**
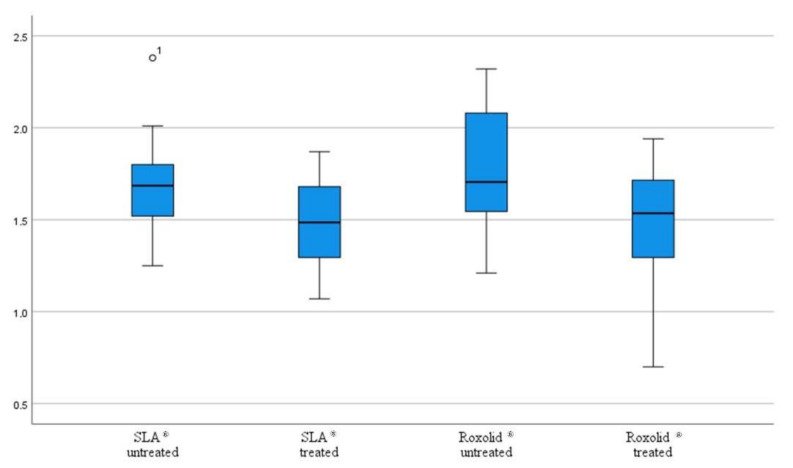
Mean roughness values of the two implants SLA^®^ and Roxolid^®^ as a boxplot. (Number 1 at the SLA® untreated shows a statistical outlier).

The individual Ra values of the untreated and treated implant thread areas are shown in Table 1 for the SLA^®^ and in Table 2 for the Roxolid^®^. A change in the Ra value for both implants could be seen at a laser energy of 160 mJ. Figure 10 shows the mean roughness values of the two implants, SLA^®^ and Roxolid^®^.

### 3.1. SLA^®^

Initially, the implant surfaces of the head and those of the left flank were affected by a laser energy of 160 mJ. A Ra value of 2.38 µm at the untreated head surface and a Ra value of 1.73 µm at the left flank surface were measured. A Ra value of 1.87 µm at the treated implant head surface and a Ra value of 1.42 µm at the treated left flank surface were measured. At even higher energies, the surface of the left flank was no longer affected.

The partial surfaces of the head, valley, and right flank showed a respective decrease in Ra values up to a laser energy of 250 mJ. The Ra value of the implant surfaces treated with 180 mJ ranged from 1.32 µm to 1.78 µm. For a laser energy of 200 mJ, the value was 1.23 µm to 1.71 µm. The largest difference was seen at an energy of 250 mJ, which had an Ra value of 1.07 µm to 1.86 µm. The mean roughness values of all treated implant surfaces showed a significant decrease compared to the untreated surfaces (*p* = 0.003).

### 3.2. Scanning Electron Microscope (SEM)

**Figure 11:**
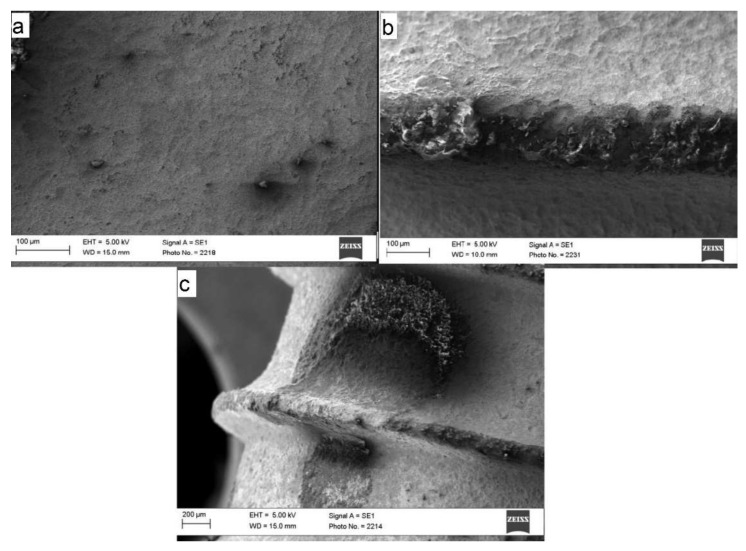
(**a**) The SLA^®^ shows a slight surface roughness when untreated. Uneven spots can be seen in the valley. (**b**) The SEM images with a laser energy of 160 mJ showed significant changes, especially at the head of the threads. Less noticeable changes were seen in the valley. (**c**) The surface could be significantly altered at the valley in tandem with the increasing energy level at 250 mJ. This resulted in severe structural changes to the surface of the implant.

SEM images with a laser energy of 160 mJ showed significant changes, especially at the head of the threads (Figure 11). The surface was also significantly removed in tandem with the increasing energy level. This can be seen from the SEM image of the treated area at an energy of 250 mJ. A treatment of up to 160 mJ and a treatment duration of up to 60 s could be recommended. The energy level could be increased up to 200 mJ because the surface remains rough despite the treatment.

### 3.3. Roxolid^®^

The implant surfaces of the valley and the flanks were not affected at a laser energy of 160 mJ. The head surface assumed a Ra value of 1.75 µm, which was 2.14 µm in the untreated implant surface. At an energy level of 180 mJ, a decrease could still only be seen at the head of the thread. The significant decrease in the Ra value could be seen at an energy of 200 mJ at the head, particularly at the flanks.

The valley of the pitch could be affected for the first time at an energy level of 250 mJ, and the mean roughness values of all treated implant surfaces showed a significant decrease compared to the untreated surfaces (*p* = 0.001).

### 3.4. SEM Analysis

**Figure 12:**
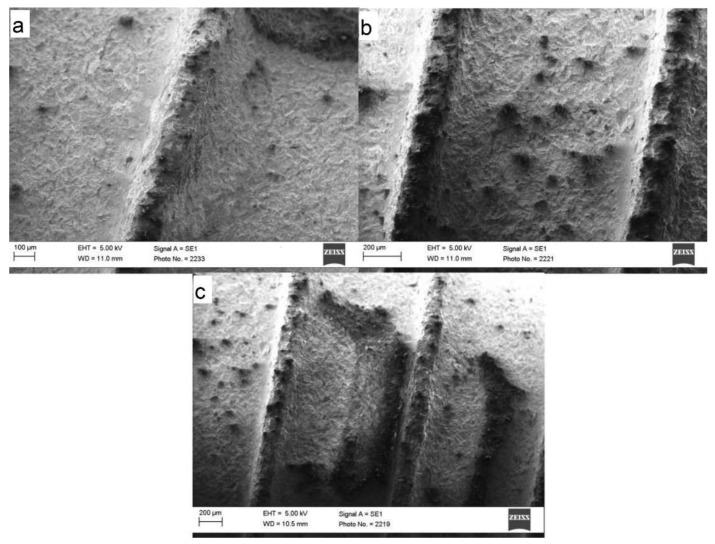
(**a**) The Roxolid^®^ shows surface roughness in isolated cases in the valley, on the flanks, and on the head. The unevenness appears somewhat more pronounced on the head. The valley seems to be plain. (**b**) The Roxolid^®^ could mostly maintain its surface topography at a level of 160 mJ. The changes in roughness and structural damage increased in the valley as well as at the flanks and the head. (**c**) At an energy level of 250 mJ, the surface properties of the implant could be significantly changed for the first time. At this energy, structural changes in the entire implant surface occurred. However, compared to the Standard Plus dental implants studied, the alterations at the surface were not severe.

SEM images (Figure 12) showed that Roxolid^®^ could maintain its surface topography at a level of 160 mJ in particular. At an energy level of 250 mJ, the surface properties of the pitch could be significantly altered for the first time. Compared to the Standard Plus dental implants studied, no distinct removal of the material from the surface was detected. The energy level could be increased up to 250 mJ due to the stability of the material.

## 4. Discussion

The literature reveals that titanium zirconium alloys show very good biocompatibility, superior corrosion resistance compared to titanium, and a strength of up to 40% higher than the strength of grade IV titanium, which makes them become a dominant implant material of the future [22]. Despite the well-described role of laser treatment in the management of peri-implantitis, the number of studies evaluating the effects of an Er:YAG laser on titanium-zirconium alloys is limited. The current study aims to clarify the effects of Er:YAG laser application on dental implant threads of titanium–zirconium alloy.

The role of Er:YAG laser therapy in the management of peri-implantitis therapy has been discussed controversially in the literature. A review by Schwarz et al. of the use of an Er:YAG laser alone found it unsuccessful in the treatment of peri-implantitis [23]. It has also been suggested that Er:YAG treatment has no influence on the attachment rate of osteoblasts on the implant surface [24]. On the other hand, Matsuyama et al. have shown that an Er:YAG laser has the ability to both remove granulation tissue and decontaminate the implant surface without changing the titanium surface [25]. They have also stated that irradiation at 100 mJ could result in distinct color changes in titanium plates, whereas 200 mJ leads to significant morphological alterations. Shin et al. have shown that the SLA^®^ implant surfaces were not altered even when Er:YAG laser energies of up to 100 and 140 mJ [26]. These findings mainly confirm the results expressed herein; however, the current study has evaluated the alterations in each component of a thread pitch separately.

The results of the current study showed that each thread pitch could present different roughness values. Therefore, the results of the experimental studies using standardized plates as samples could differ from the results obtained from experimental designs using dental implants as samples. The current study also showed that the heads of the threads showed an inclination of alteration even at lower energy levels in both implant types, compared to the flanks and the valley of the pitch. During the clinical application, it is also not possible to irradiate each area equally; thus, the angulation of the laser probe could vary depending on anatomical structures. Additionally, the differences in implant macro-topography in terms of thread design could result in different alterations despite laser irradiation at a similar energy level.

A recent study by Tan et al. investigated the generation of titanium and zirconium particles from titanium and zirconium implant surfaces during the Er:YAG laser decontamination process and evaluated morphological changes to the implant surfaces as a result of the laser irradiation [27]. Despite the lack of topographical alterations, even at an energy level of 40 mJ, titanium and zirconia particles were detected after laser irradiation on the implants. Therefore, the biological aspects of the laser treatment of titanium-zirconium alloys should be furtherly studied with in-vivo models, as the nanoparticles could result in cytotoxicity, especially in preosteoblastic cells [28]. Similarly, in order to gain an understanding of the clinical aspects, cell proliferation characteristics and bacterial colonization on irradiated Roxolid^®^ implants should be further investigated.

In the literature, despite promising results, there is a lack of scientific evidence regarding the clinical efficiency of Er: YAG irradiation in peri-implantitis treatment on Ti surfaces. A recent pilot study indicated a reduction in probing depth [29]. Similarly, Li et al. have also shown that Er:YAG lasers offer health benefits to patients with peri-implantitis and can effectively reduce probing depth and gingival recession. All of the articles have clearly mentioned the need for a larger sample size and longer follow-up to confirm whether Er: YAG laser irradiation provides additional clinical benefits for peri-implantitis regenerative therapy due to the variations in clinical results. This might be attributed to the differences in implant geometry and thread design, which could influence the ablative effects of laser irradiation, as shown herein [20].

The main goal of adjunctive peri-implantitis treatment is the decontamination of the implant surface. Due to its antimicrobial effects, cold atmospheric plasma (CAP) has gained popularity in peri-implantitis therapy in recent years. However, the effects of CAP treatment on titanium-zirconium alloy have not been evaluated yet. Considering the well-defined decontamination properties of Er:YAG irradiation on titanium surfaces, a comparative assessment of both CAP and Er:YAG in peri-implantitis therapy might be of interest [25].

Finally, the implant characteristics have a major impact on the absorption of laser light. In this study, the titanium–zirconium alloy of the Roxolid^®^ seems to be sturdier and more resistant. However, changes in surface roughness can be expected from a laser energy of 160 mJ. The implant surface should not be destroyed with a laser, which is why the precise energy setting is important. Schwarz et al. did not find significant changes in the roughness of different titanium surfaces by using an Er:YAG laser with a 100 mJ/pulse at 10 Hz for 60 s [24]. Kreisler et al. showed alterations on SLA surfaces using an Er:YAG laser with a 120 mJ/pulse for 5 s in a single spot [30]. Shin et al. concluded that the exact energy to detoxify and decontaminate the surface without significant alterations is different for the respective implant surfaces: 100 mJ for anodically oxidized surface implants and 140 mJ for SLA [26]. Deppe et al. reported that different implant characteristics result in different thermal conductivity. The specific thermal capacity of titanium is higher than that of zirconium oxide. In this way, heat accumulates in the ceramic material and is dissipated to a lesser extent than in titanium [31].

A common complication of implant surface changes is corrosion. Ultimately, no further analyses were conducted regarding the resistance of the implants, but Sikora et al. explained that titanium-zirconium alloys were significantly more resistant to corrosion than titanium alone [32]. One problem is the change in the coating, which can also be seen in the SEM in this study. Morphological features seen with the SEM included the chipping of the coating at too high energy values, which could be seen from 160 mJ.

Furthermore, a recent article has evaluated the effects in terms of surface temperature alterations of 2780 nm Er,Cr:YSGG and 940 nm diode laser irradiation on four dental implant surfaces, including Roxolid^®^ [33]. It has also been shown that Roxolid^®^ disclosed droplet-formed areas of Ti in the range of 2–10 μm on the prominent parts of the threads. At higher output power, more extensive alterations, such as areas of melting TiO_2_ particles, a totally ablated oxide layer, and melted titanium droplets, were observed on titanium surfaces. Similar to the results expressed by Fahlstedt et al., the current study has also shown that the titanium-zirconium alloy of Roxolid^®^ showed a more stable structure after Er: YAG irradiation compared to titanium implants, which might be attributed to its melting point of 3000°. This could also be explained by its superior energy absorption in particular. In the current study, thermal changes on the irradiated implant surfaces have not been investigated. However, it is well known that, among all dental lasers, the Er:YAG laser has the highest absorption into water, which minimizes the thermal effects on the surrounding tissues during irradiation [34]. This fact might be the subject of a future study on Roxolid^®^ implants.

## 5. Conclusions

Er:YAG irradiation could result in changes in the surface topography of Roxolid^®^ implants, but unlike the SLA-Standard^®^ dental implant, the energy level required for the appearance of alterations was significantly higher.

The alloy properties of Roxolid^®^ confirm the manufacturer’s statement in terms of stability and could offer advantages in peri-implantitis management if decontamination has been selected. However, as a part of a resective strategy, smoothening a Roxolid^®^ implant surface requires a significantly higher energy level compared to SLA-Standard^®^ dental implants. This point could be a decisive factor in implant selection for dental implant candidates with increased peri-implantitis risk. It should also be kept in mind that the same energy level results in different alterations at the thread pitch, which could influence the decision on the implant system regarding the implant geometry.

Further in-vivo studies would be beneficial to clarify the clinical aspects of the Er:YAG irradiation on Roxolid^®^ implants, as well as problems like corrosion, returning peri-implantitis, and implant loss have to be analyzed.

## Figures and Tables

**Table 1:** Illustration of the treated and untreated implant surfaces of the SLA^®^. Shown are the implant thread areas “3” to “6” where a change in roughness (*) was measured due to treatment with an Er:YAG laser. The values were given in µm. No changes could be observed in implant thread areas “1” and “2” at 120 and 140 mJ, respectively.

Measuring Range	Head	Valley	Flank (R)	Flank (L)
Thread “3” untreated	2.38	1.52	1.25	1.73
Thread “3” treated with 160 mJ	1.87 *	1.53	1.18	1.42 *
Thread “4” untreated	2.01	1.88	1.52	1.4
Thread “4” treated with 180 mJ	1.78 *	1.65 *	1.32 *	1.35
Thread “5” untreated	1.71	1.78	1.59	1.64
Thread “5” treated with 200 mJ	1.33 *	1.27 *	1.23 *	1.71
Thread “6” untreated	1.66	1.74	1.5	1.82
Thread “6” treated with 250 mJ	1.07 *	1.44 *	1.52	1.86

**Table 2:** Illustration of the treated and untreated implant surfaces of the Roxolid^®^. Shown are the implant thread areas “3” to “6” where a change in roughness (*) was measured due to treatment with an Er:YAG laser. The values were given in µm. No changes could be observed in implant thread areas “1” and “2” at 120 and 140 mJ, respectively.

Measuring Range	Head	Valley	Flank (R)	Flank (L)
Thread “3” untreated	2.14	1.65	1.69	1.62
Thread “3” treated with 160 mJ	1.75 *	1.65	1.59	1.50
Thread “4” untreated	2.32	1.79	1.32	1.55
Thread “4” treated with 180 mJ	1.78 *	1.68	1.23	1.48
Thread “5” untreated	2.1	2.08	1.54	1.22
Thread “5” treated with 200 mJ	1.45 *	1.99	1.22 *	1.01 *
Thread “6” untreated	2.08	1.88	1.72	1.21
Thread “6” treated with 250 mJ	1.84 *	1.58 *	1.58 *	0.7 *

## Data Availability

Not applicable.
